# Brown Adipose Tissue Activation Is Inversely Related to Central Obesity and Metabolic Parameters in Adult Human

**DOI:** 10.1371/journal.pone.0123795

**Published:** 2015-04-20

**Authors:** Qidi Wang, Min Zhang, Min Xu, Weiqiong Gu, Yun Xi, Lu Qi, Biao Li, Weiqing Wang

**Affiliations:** 1 Shanghai Key Laboratory for Endocrine Tumors, Rui-Jin Hospital, Shanghai Jiao-Tong University School of Medicine, Shanghai, China; 2 Department of Endocrine and Metabolic Diseases, Shanghai Clinical Center for Endocrine and Metabolic Diseases, Rui-Jin Hospital, Shanghai Jiao-Tong University School of Medicine, Shanghai, China; 3 Department of Nuclear Medicine, Rui-Jin Hospital, Shanghai Jiao-Tong University School of Medicine, Shanghai, China; 4 Department of Nutrition, Harvard School of Public Health, Boston, Massachusetts, United States of America; The Ohio State University, UNITED STATES

## Abstract

**Background:**

Recent studies have shown that adult human possess active brown adipose tissue (BAT), which might be important in affecting obesity. However, the supporting evidence on the relationship between BAT and central obesity and metabolic profile in large population based studies is sparse.

**Methodology/Principal Findings:**

We studied 4011 (2688 males and 1323 females) tumor-free Chinese adults aged 18-89 for BAT activities, visceral/subcutaneous fat areas (VFA/SFA), waist circumferences (WC) and metabolic parameters. We found that the prevalence of BAT was around 2.7% in our study participants, with a significant sexual difference (5.5% in the females vs. 1.3% in the males; p<0.0001). BAT detection was increased in low temperature and declined in elderly subjects. The BAT positive subjects had lower BMI (P<0.0001), less SFA (P<0.01), VFA (P<0.0001), WC (P<0.0001), lower fasting glucose and triglyceride levels (both P<0.01) and increased HDL cholesterol concentrations (P<0.0001), compared with the BAT negative subjects. Robust logistic regression revealed that after adjustment for covariates (including age, sex, BMI, VFA, SFA and WC), age and BMI in the males (0.92 [95%CI, 0.88-0.96] and 0.84 [95% CI, 0.75-0.96], both P ≤0.008) while age and VFA in the females (0.87 [95%CI, 0.83-0.91] and 0.98 [95%CI, 0.97-0.99], respectively, P<0.05) were independently associated with detectable BAT.

**Conclusions/Significance:**

Our data suggest that decreased amount of active BAT might be associated with accumulation of visceral fat content and unfavorable metabolic outcomes.

## Introduction

It was widely considered that brown adipose tissue (BAT) is present only in fetuses and infants and diminishes in adults [1]. In the late 1990s, acceptance was gradually gained among radiologists that the symmetrical regions in the neck and chest with high ^18^F-fluoro-deoxy-glucose uptake in PET/CT scans represented brown adipose tissue in adults, but their nature remained unknown for years [2]. Until 2009, several studies have clearly demonstrated that adults possessed metabolically active brown fat detected by PET/CT imaging [3,4,5]. Recent data suggest that there are two distinct types of brown fat: classical brown fat and UCP1-positive cells that emerge in white fat, called “beige” adipose cells [6,7]. Based on biopsy material from neck fat in healthy human, Cypess et al reported that human samples share many similarities with classical rodent BAT [8], while Spiegleman’s group using their brown and beige fat cells lines, argued that those brown-like cells in adult humans are much more similar to murine beige fat cells [9]. A major task now is to understand the contribution that this tissue makes to overall metabolic homeostasis in adult humans.

In rodents, mice genetically engineered to have increased activity of BAT, beige fat or both resist weight gain and display improvements in glucose tolerance and insulin sensitivity [10,11]. In adult human, it is confirmed by many groups that the amount of BAT is inversely correlated with body mass index (BMI), indicating a potential role of BAT in regulation of body weight [3,4,5,12]. Possible contribution of BAT to metabolism has also been suggested [13]. Recently, a prospective crossover study on 5 healthy men found that BAT recruitment induced by temperature acclimation during 4-month period enhanced their diet-induced thermogenesis and post-prandial insulin sensitivity [14]. Activated BAT might also control triglyceride clearance, and thus exert beneficial effect on metabolism [15]. It is well-known that visceral fat has a close association with metabolic disease, such as hypertension, insulin resistance, type 2 diabetes, dyslipidemia and coronary heart disease [16,17,18,19,20]; whereas the subcutaneous fat does not (and may actually be preventative) [14]. Unfortunately, the link between BAT activity, visceral fat accumulation and metabolic risk in large population has not yet been well defined. Our previous study has shown that BAT was inversely related with central obesity parameters (visceral fat areas, visceral/total fat areas, waist circumferences), but not with subcutaneous fat areas [21]; however the sample size was too small to draw a conclusion.

In the present study, by using consecutive ^18^F-fluorodeoxyglucose (^18^F-FDG) PET/CT whole-body scans and comprehensive fat area measurements, we extended our research to assess the relations of BAT, body weight, subcutaneous and internal abdominal (visceral) fat, and metabolic markers in 4011 tumor-free Chinese adults.

## Materials and Methods

### Ethics statement

This study was approved by the Institutional Review Board of the Rui-jin Hospital Affiliated to Shanghai Jiao-Tong University School of Medicine and was in accordance with the principle of the Helsinki Declaration II.

### Subjects

The application of PET/CT for the detection of tumors was introduced in the Department of Nuclear Medicine of Shanghai Ruijin Hospital in 2007 and soon gained increased use for cancer surveillance in routine medical examination. The individuals from routine medical examination (less than 3% tumor occurrence) constitute approximately 70% of all subjects undergoing PET/CT scans in the hospital each year. A total of 4082 healthy asymptomatic adult subjects underwent consecutive whole-body ^18^F-FDG PET/CT scans between May 2007 and February 2010 for voluntary cancer screening were evaluated and finally 4011 subjects (2688 males and 1323 females) free of malignant tumors were included in the present study. The numbers of study subjects in each month were listed below: Jan 277, Feb 149, Mar 265, Apr 278, May 343, June 407, July 406, Aug 326, Sep 313, Oct 386, Nov 436, Dec: 425. The written informed consent was obtained from each participant for allowing to do research on their available data.

Data on age, sex, height, weight were obtained from all the subjects. 2222 (1549 males and 673 females) subjects have performed the examinations of fasting glucose, triglyceride, total cholesterol, high density lipoprotein (HDL) cholesterol, low density lipoprotein (LDL) cholesterol, within 1 week before or after PET/CT scans. Outdoor temperatures in Shanghai for the dates of scans were obtained from Shanghai Meteorological Bureau.

### PET/CT imaging

All of the subjects were studied after an overnight fast. ^18^F-FDG PET/CT scans were performed with a Discovery STE16 integrated PET/CT scanner (GE Medical Systems) as previously described [21]. PET and CT images were co-registered and analyzed with Volume Viewer software. The activity of BAT was quantified by standardized uptake values (SUV[g/ml]), defined as the activity per milliliter within the region of interest divided by the injected dose in megabecquerels per kilogram of body weight, which is an automatic method based on Volume Viewer software [22]. BAT was considered positive if there were areas of tissue that were more than 4 mm in diameter, had the CT density of adipose tissue (−250 to −50 Hounsfield units), and had a maximal SUV of ^18^F-FDG of at least 2.0 g per milliliter as a cutoff value of the lower boundary of BAT activity [3,21].

### Assessment of visceral/subcutaneous fat areas and waist circumferences

Abdominal fat distributions in 3498 subjects (2336 males and 1162 females) were examined at the umbilicus level in the supine position using CT, according to the previously described procedure [23]. The intra-abdominal visceral fat areas (VFA), subcutaneous fat areas (SFA), and waist circumferences (WC) were measured with image analysis software package (Fat scan, N2 system, Osaka, Japan). Overweight and obesity were defined as having a BMI of 25–30 kg/m2 and ≥30 kg/m2 according to WHO criteria [24]. Central obesity was defined as waist circumference ≥ 90 cm in men and ≥ 80 cm in women [25].

### Statistical analyses

Statistical analyses were performed with SAS 9.2 (SAS Institute, Cary NC). All continuous parameters were summarized by means ± SD and were compared between study groups with the use of analysis of variance (ANOVA). For skewness distribution data, median and interquartile range was used and the difference was examined by using rank sum tests. The roles of SFA, VFA and WC, as predictors of positive BAT were tested by using the univariate and backward multivariate logistic regression models after adjustment for age and BMI, in a sex- and temperature-specific pattern.

To test the presence of BAT by metabolic markers, we grouped the patients as tertiles for fasting glucose, triglyceride and cholesterol levels; the significance of linear trends across the tertiles was tested by assigning each participant the median value for the third and modeling this value as a continuous variable. Odds ratios and 95% confidence intervals were estimated as measures of the magnitude of the associations. The P-values reported were two-sided. A P-value of less than 0.05 indicated statistical significance.

## Results

2688 male and 1323 female subjects who had performed the PET-CT scans were included in the present study. The average outdoor temperatures when the subjects underwent the PET-CT scans were comparable between the male and the female subjects. As compared with the female subjects, the male subjects were slightly older (p = 0.04) and had higher BMI levels (p<0.0001) (Table 1). Consistent with their difference in BMI, the total fat areas, visceral fat areas and waist circumferences in the males were higher than those in the females (all P ≤ 0.04, Table 1). On the contrary, the subcutaneous fat areas in the males were significantly lower than those in the females (p<0.0001, Table 1). With respect to the metabolic profile, the male subjects had significantly higher levels of fasting glucose, triglycerides, total cholesterol and lower HDL cholesterol concentrations than the female subjects (all p<0.05, Table 1). Moreover, LDL cholesterol levels also tended to be higher in male subjects, but did not reach statistical significance (Table 1). As expected, the prevalence of obesity and diabetes in the male subjects was greater than that in the females (both P < 0.0001, Table 1).

**Table 1:** Characteristic of the study subjects.

Subject profile	Male subjects	Female subjects
**N**	**2688**	**1323**
Age (Years)	47.1 (42.1–54.1)	46.1 (41.1–54.1)*
Height (cm)	171.8 ± 5.1	160.8 ± 4.8†
Body Weight (Kg)	73.6 ± 10.1	57.8 ± 8.7†
BMI (Kg/m^2^)	24.9 ± 3.0	22.3 ± 3.1†
Outdoor temperature (°C)	18.3 ± 8.6	18.0 ± 8.9
**Fat Areas (cm** ^**2**^ **) N**	**2336**	**1162**
Total	230.7 ± 87.6	223.8 ± 95.3*
Visceral	103.5 ± 48.9	61.5 ± 37.8†
Subcutaneous	127.8 ± 53.1	163.7 ± 72.9†
Waist circumferences (cm)	88.7 ± 9.2	83.3 ± 9.7†
**Metabolic profile (mmol/L) N**	**1549**	**673**
Fasting glucose	5.11 ± 1.06	4.90 ± 0.97†
Triglycerides	1.73 [1.18–2.59]	1.03 [0.58–1.48] †
Cholesterol	4.95 ± 0.96	4.86 ± 0.96*
HDL	1.20 ± 0.30	1.48 ± 0.33†
LDL	3.01 ± 0.81	2.94 ± 0.82
**Obesity group, n (%)**		
Non-obese (BMI < 25 kg/m^2^)	1411 (52.48)	1092 (82.54)
Overweight (30>BMI≥25 kg/m^2^)	1158 (43.08)	204 (15.42)
Obesity (BMI ≥ 30 kg/m^2^)	119 (4.43)	27 (2.04) †
**Diabetes, n(%)§**	73 (4.71)	12 (1.78) †

All continuous parameters were summarized by means ± standard deviation (SD) or number (proportions). For skewness distribution data, median and interquartile range was used. P values were calculated from analysis of variance for continuous variable, chi-square tests for categorical variable or rank sum tests for skewness distribution variable:

*p<0.05,

^†^p<0.0001.

^§^ Analysis was performed in 2222 subjects. Diabetes was defined as fasting glucose ≥ 7.0 mmol/L.

**Fig 1:**
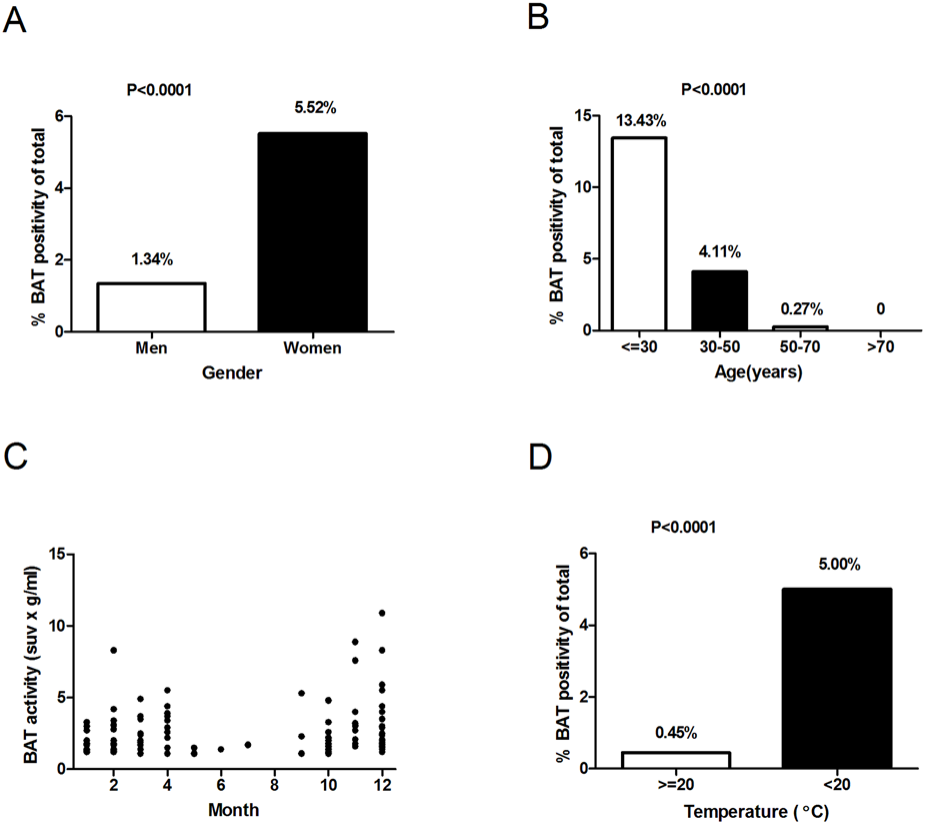
Correlation between the prevalence of badipose tissue and temperature, age and gender. Panel A shows the prevalence of detectable BAT in men and women. Panel B shows the percentage of patients in different age ranges that had detectable BAT. In panel C, for the patients with detectable BAT, the activity of BAT in grams times the mean standardized uptake value (SUV) in grams per milliliter was shown in each month during a 3 year period from May 2007-Feb 2010. In Panel D, outdoor temperatures in Shanghai for the dates of scans were obtained and the percentage of subjects with detectable BAT in different temperature ranges was determined. A univariate analysis was used to assess the significance of differences in the percentages with the use of a chi-square test.

The prevalence of BAT was significantly higher in female than that in male subjects (5.52% vs. 1.34%, P < 0.0001, table 1, Fig 1A). The detection rate of BAT was 13.43%, 4.11%, 0.27% and 0% in subjects less than 30, 30–50, 50–70 and more than 70 years old, respectively (Fig 1B). The average age was significantly lower in BAT positive subjects both in the male and the female subjects (Table 2). The probability of the detection of BAT presented seasonal variation (Fig 1C), i.e. being lowest for subjects measured in summer (0.18%, June-August), moderate in spring (2.71%, March-May) and autumn (2.11%, September-November) and highest in winter (6.93%, December-February, P<0.001). It has been reported that a relative low temperature at 19–21°C may be more appropriate to detect BAT [12,26]. Indeed, the prevalence of BAT was significantly higher when outdoor temperature was below than above 20°C (5.00% vs. 0.45%, P < 0.0001, Fig 1D). Moreover, mean outdoor temperature was significantly lower in subjects bearing detectable BAT in both the males and the females (P<0.0001, Table 2)

**Table 2:** Comparison of body fat areas between subjects bearing detectable (+) and undetectable (-) amounts of BAT by gender.

	Male subjects		Female subjects	
**Brown adipose tissue**	**+**	**-**	**+**	**-**
N	36	2652	73	1250
BAT prevalence, n (%)	36 (1.34%)		73 (5.52%)*	
Age (Years)	41.1(36.6–46.1)	47.1(42.9–54.1)*	38.1(33.0–42.1)	47.1(42.0–55.1)*
Height (cm)	172.0±4.7	171.8±5.1	161.6±4.5	160.8±4.8
Body Weight (Kg)	67.5±8.7	73.6±10.1†	53.3±6.9	58.0±8.7*
BMI (kg/m^2^)	22.8±2.7	24.9±3.0*	20.4±0.3	22.4±0.09*
Temperature (°C)	10.1±7.1	18.5±8.6*	10.3±6.3	18.5±8.9*
**N**	33	2303	64	1098
**Fat Areas (cm** ^**2**^ **)**					
Total	182.9±89.3	231.3±87.4†	163.4±66.3	227.5±95.6*
Visceral	81.3±49.7	103.8±48.9†	36.7±25.3	63.0±37.9*
Subcutaneous	100.1±44.9	128.2±53.1†	126.4±46.8	165.8±73.3*
Waist circumferences (cm)	83.3±8.8	88.7±9.2†	77.3±6.6	83.7±9.8*

All continuous parameters were summarized by means ± SD. For skewness distribution data, median and interquartile range was used. P values were calculated from analysis of variance for continuous variable, chi-square tests for categorical variable or rank sum tests for skewness distribution variable:

*p<0.05,

^†^p<0.0001.

**Fig 2:**
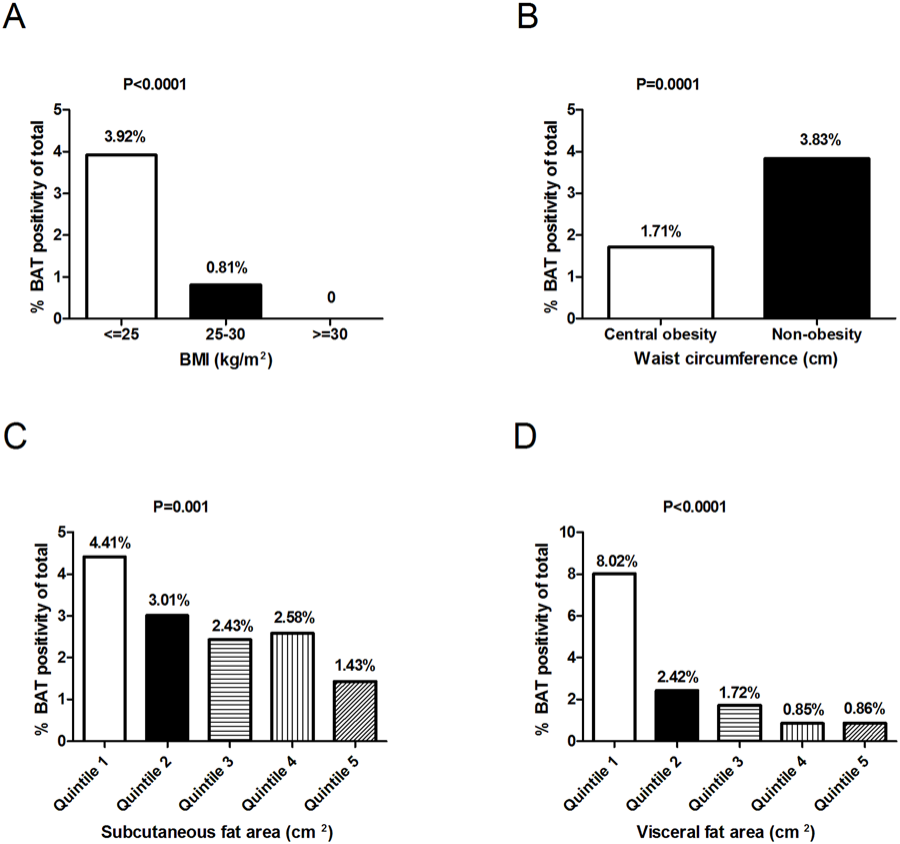
The prevalence of brown adipose tissue was inversely related with adiposity. Body-mass-index (the weight in kilograms divided by the square of the height in meters, panel A), Waist Circumferences (panel B), Subcutaneous Fat Areas (Panel C), Visceral Fat Areas (Panel D). The percentage of patients in each group (shown as BMI<25, 25~30, ≥30; central obesity and non-obesity which were defined from waist circumferences; and quintiles of subcutaneous and visceral fat areas) who had detectable BAT was shown, and a univariate analysis was used to assess the significance of differences in the percentages with the use of a chi-square test.

The detectable BAT was more frequent in non-obese subjects defined by either BMI or waist circumference (Fig 2A and 2B). The presence of BAT was steeply decreased along with the increasing subcutaneous fat areas (Fig 2C) and especially visceral fat areas (Fig 2D). Both in the males and the females, the BAT positive subjects had lower BMI and less subcutaneous fat areas, visceral fat areas and waist circumference, compared with the BAT non-detectable subjects (P<0.01, Table 2). Consistently, similar results were obtained using another central obesity parameter, waist-to-height ratio [27]. Both in men and in women, the waist-to-height ratio was significantly lower in subjects with detectable BAT (0.48 ± 0.05 vs. 0.52 ± 0.05 in men, P = 0.0004; 0.48 ± 0.04 vs. 0.52 ± 0.06 in women, P <0.0001).

To exclude the possibility that the inverse correlation between BAT and obesity is simply due to a better insulated characteristic of the obese people, we performed logistic regression analysis to predict the presence of positive BAT in subjects with low outdoor temperature (<20°C, cold exposed) that was sufficiently cold to activate BAT [12]. This could also minimize the potential residual confounding by outdoor temperature. Unadjusted analysis showed that both in the males and in the females, age, BMI, waist circumference were significantly and inversely related to BAT positivity (Table 3). In the models adjusting for covariates (including age, BMI, VFA, SFA, WC), age and BMI in the male subjects (ORs = 0.92 [95%CI, 0.88–0.96] and 0.84 [95% CI, 0.75–0.96], both P ≤ 0.008) while age and visceral fat areas in the female subjects (Ors = 0.87 [95%CI, 0.83–0.91] and 0.98 [95%CI, 0.97–0.99], respectively, P<0.05) were independently associated with detectable BAT (Table 3).

**Table 3:** The unadjusted and adjusted ORs and 95% CI from the logistic regressions predicting the likelihood of positive BAT in the subgroup with low outdoor temperature (<20°C).

	Male subjects		Female subjects	
	Unadjusted OR (95% CI)	Adjusted OR (95% CI)	Unadjusted OR (95% CI)	Adjusted OR (95% CI)
Age (years)	0.90 *(0.86–0.94)	0.92 †(0.88–0.96)	0.87 *(0.83–0.89)	0.87 *(0.83–0.91)
BMI (kg/m^2^)	0.80 †(0.71–0.90)	0.84† (0.75–0.96)	0.75 *(0.67–0.83)	-
WC (cm)	0.93 *(0.90–0.97)	-	0.93 *(0.90–0.97)	-
SFA (cm^2^)	0.99(0.99–1.00)	-	0.99* (0.98–0.99)	-
VFA (cm^2^)	0.99(0.99–1.00)	-	0.96 *(0.95–0.98)	0.98 ‡(0.97–0.99)

Data are odds ratios, 95% confidence interval. The backward multivariate logistic regression analysis was used to evaluate the ORs for positive BAT:

*P<0.0001,

^†^P<0.001,

^‡^P<0.05.

WC, Waist Circumferences; SFA, subcutaneous fat areas; VFA, visceral fat areas

**Fig 3:**
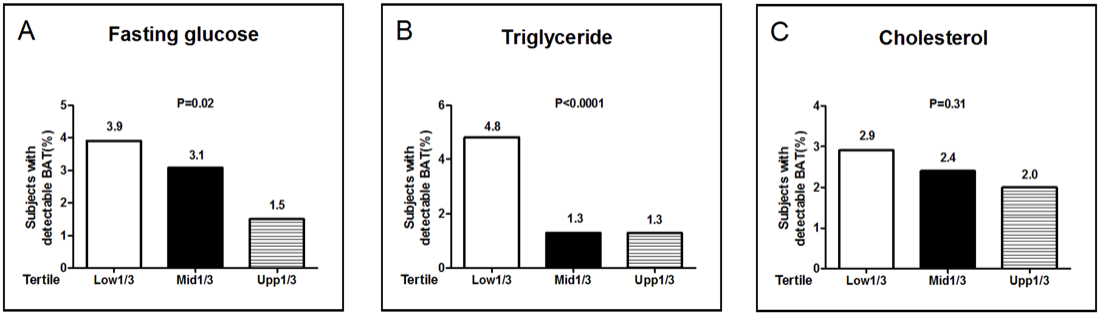
The prevalence of brown adipose tissue was nversely related with metabolic parameters. Fasting plasma glucose (Panel A), triglycerides (Panel B) and total cholesterol (Panel C) levels were divided into thirds. The percentage of patients in each subgroup who had detectable BAT was shown, and the P values for trend were calculated by using Mantel-Haenszel Chi-Square test.

We further compared BAT prevalence across the tertiles of metabolic parameters, and found that BAT was most frequently detected in subjects in the lowest tertile of fasting plasma glucose (Fig 3A) and triglyceride (Fig 3B). We also examined the metabolic parameters in people bearing detectable and undetectable amounts of BAT. We found that both the mean fasting glucose (4.6±0.5 vs. 5.0±1.0, P<0.0001) and triglyceride (0.92 (0.70–1.37) vs. 1.51 (1.01–2.29), P<0.0001) levels were significantly lower, whereas the average HDL cholesterol concentrations (1.44 ± 0.35 vs. 1.28 ± 0.33, P = 0.0002) were significantly higher in those bearing detectable BAT. Total cholesterol (4.74 ± 0.92 vs. 4.92 ± 0.95, P = 0.17) and LDL cholesterol (2.80 ± 0.82 vs. 2.99 ± 0.81, P = 0.078) concentrations tended to be lower in the BAT positive populations, but the differences were not statistically significant.

## Discussion

The current obesity epidemic and associated increased incidence of diabetes, hypertension, hyperlipidemia, cancer, and other disorders has motivated strong interest in the function of fat in affecting obesity [28]. Brown adipose tissue (BAT) is a key site of thermogenesis in mammals that has for many decades been considered as an attractive target to promote weight loss. In rodents, it is found that BAT had a central role in protecting mice from diet-induced obesity, while ablation of BAT reduced energy expenditure and increased obesity in response to high-fat diets [29,30]. In humans, multiple studies have shown that a higher degree of obesity is associated with less brown adipose tissue, suggesting potential roles of BAT in regulation of body fat contents[3,4,5,12]. The current interest in BAT has centered on its capacity to counteract metabolic disease, including obesity and type 2 diabetes. Several groups have recently shown that adults with active BAT might have favorable metabolic profiles [31,32]. However, the supporting evidence that BAT is correlated with central obesity and related metabolic risk in large population based studies is sparse.

In the present study of 4011 tumor-free Chinese adults, we observed active BAT located in the cervical-supraclavicular region and the paravertebral region, which have been previously confirmed as the major sites for BAT in adult human by immunohistochemistry with UCP-1 expression [4,5,12]. We found that the prevalence of BAT was around 2.7% in Chinese subjects, with a clear sexual preference in the females (5.5% vs. 1.3% in the males). The sex-difference in BAT occurrence (9.94% in women and 2.48% in men) remained when outdoor temperature was below 20°C. A decline in detection rate of brown adipose tissue was observed with increasing age. The BAT detection was significantly increased in lower temperature and a clear seasonal variation in BAT prevalence was observed, being highest in winter and lowest in summer. The BAT prevalence observed in our study is consistent with several earlier retrospective studies performed in Europe, Asia and Australia reporting a BAT prevalence of 2 to 7% [3,31,33,34,35,36]. We compared our result with the findings reported by Cypess et al that used the similar BAT quantification method [3]. Considering that the mean outdoor temperature in Shanghai during the time of the study was 6.3°C in winter and 27.8°C in summer, whereas below 1.6°C and 23.8°C in Cypess study [3]; it is quite likely that the slightly lower BAT prevalence in our study (2.7% vs 5.4% in Cypess data) is attributable to the higher local temperature. Moreover, the difference of genetic background between the Chinese and American population may also contribute to the diversity in BAT prevalence. A recent prospective, case-controlled observational study found that south Asian populations had lower BAT volumes than white Caucasians, which might underlie their high susceptibility to metabolic disturbances, such as obesity and type 2 diabetes [37]. It is notable that the values from our and other retrospective studies should be considered as minimal (physiological) estimate of BAT prevalence in an un-stimulated state. Indeed, the BAT activity can be further activated. For example, several previous studies have shown that BAT detection could reach up to 30–100% with cold stimulation [4,5,12,32,38,39].

Early evidence for BAT as a tissue affecting adiposity came from studies of animals with surgically denervated interscapular BAT [40] or transgenic mice with 60–70% reduction in BAT mass [41,42]: these animals accumulated abnormal amounts of body fat. Consistent with several recent studies in adult humans [3,4,12], our data showed an inverse relation between active BAT and overall adiposity. In the present study, we found that the probability of detecting visible BAT was steeply decreased from non-obese (BMI<25, 3.92%) to overweight (BMI 25–30, 0.81%) and obese subjects (BMI≥30, 0%). Interestingly, a detailed fat area analysis showed that central obesity parameters, i.e. visceral fat areas, waist circumferences and waist-to-height ratio were all significantly lower in subjects with detectable BAT. Moreover, subjects without central obesity had more than 2-fold higher BAT detection rate than those with central obesity. These results are consistent with two recent findings by Japanese groups: Nakayama K et al observed UCP1 exhibited significant associations with VFA and VFA adjusted for BMI from winter to spring [43]and the group of Saito reported that subjects with detectable cold-activated BAT exhibiting lower adiposity-related parameters such as the BMI, body fat mass and abdominal fat area [32]. Using logistic regression analysis, we also noted that after adjusting for covariates, the association between BAT and visceral fat were more evident in the females than in the males. Given the fact that a 3-fold higher prevalence of BAT was found in women, one might expect a more pronounced decrease in visceral fat content in the females. Indeed, marked sexual dimorphism in regional fat composition was apparent in our subjects: women have less abdominal fat (visceral fat areas and waist circumferences as indices) than men. The difference in body fat distribution between men and women has been partly accounted by sex-associated hormones, such as estrogen [44,45]. Our data suggest an alternative explanation: the difference of BAT activation due to estrogen levels between men and women [46] might also determine their differences in body fat distribution. However, we should keep in mind whether the sex-difference in BAT detection in men and women observed in all retrospective studies represents a true sex dimorphism; since a similar cold-activated BAT detection was observed in men and women, where BAT would be maximally activated [12,39]. Thus our hypothesis needs to be further confirmed in population under cold-stimulated conditions. It is currently difficult to distinguish whether a low BAT activity caused obesity, or, vice versa. However, in animal models, obesity could be induced by BAT specific protein uncoupling protein 1 (UCP1) ablation [47] and UCP1 deficiency increases susceptibility to diet-induced obesity with age [48].

In the present study, the subjects bearing detectable BAT had lower levels of fasting glucose and triglycerides and higher HDL cholesterol levels. Moreover, the likelihood of having active BAT was higher in subjects who were in the bottom tertile for fasting glucose and triglyceride levels than those in the top tertile. The inverse relation between active BAT and visceral fat content may partly account for the difference of metabolic outcomes. In addition to its influence on visceral fat content, BAT may affect glucose and lipid disposal. BAT combusts a mixture of lipid and carbohydrate substrate when it is chronically active [49], and therefore may affect homeostasis of these metabolites. Moreover, activated BAT takes up and metabolizes lipid from the bloodstream and controls triglyceride clearance and thus have beneficial effect on metabolism [15]. The beneficial effect of brown and beige fat against metabolic disturbance go beyond obesity is also supported by two recent studies: improvement in glucose tolerance seems disproportional to the modest effects on body weight in transgenic Prdm16 mice and irisin-treated mice with selectively increased beige fat activity [50]. Unfortunately, our study was underpowered to assess the relation between the metabolic markers and BAT activity. Future studies with larger sample size are warranted to test the relation between BAT and risk of metabolic disorders such as diabetes mellitus, insulin resistance and hyperlipidemia.

This is one of the few studies to date to estimate the BAT prevalence and its metabolic effects in Chinese healthy population. Furthermore, the comprehensive measurements of central fat areas allowed us to determine correlation between BAT and central obesity. However, the present finding shared common limitations in most cross-sectional studies in this field: the low detection of active BAT at un-stimulated state and the high cost of the invasive PET/CT technique limited the sample size to further assess the relation between BAT and metabolic risk; and to make causal inference. Large, cross-sectional studies are warranted to evaluate potential causal roles of BAT activation in relation to obesity, insulin resistance and metabolic disorders.

In summary, our results lend support to the presence of active BAT in Chinese adults with similar prevalence as other populations such as the Whites. We showed inverse associations of BAT with BMI and visceral adiposity parameters, i.e. visceral fat areas and waist circumference. Compared to men, women may possess greater amount of BAT, which have more pronounced impact on reducing visceral fat content. Our data also support a possible protective effect of BAT on glucose and lipid homeostasis.
